# Grape Seed Extract Positively Modulates Blood Pressure and Perceived Stress: A Randomized, Double-Blind, Placebo-Controlled Study in Healthy Volunteers

**DOI:** 10.3390/nu13020654

**Published:** 2021-02-17

**Authors:** Christiane Schön, Pietro Allegrini, Karin Engelhart-Jentzsch, Antonella Riva, Giovanna Petrangolini

**Affiliations:** 1 BioTeSys GmbH, Schelztorstr. 54–56, 73728 Esslingen, Germany; k.engelhart@biotesys.de; 2 Research and Development Department, Indena SpA, 20139 Milan, Italy; pietro.allegrini@indena.com (P.A.); antonella.riva@indena.com (A.R.); giovanna.petrangolini@indena.com (G.P.)

**Keywords:** grape seed extract, Enovita^®^, proanthocyanidins, HUVEC, blood pressure, healthy volunteers

## Abstract

It is well established that maintaining healthy blood pressure is fundamental in order to avoid disorders to the heart and blood vessels. In prevention, and alongside pharmacological therapy, the use of natural substances has been proven to be extremely helpful for pre- and mild hypertensive subjects. Our study was therefore focused on the effects, both in vitro and in humans, of a grape seed extract, Enovita (GSEe), a highly standardized extract in polyphenols of *Vitis vinifera* L. The in vitro human umbilical vein endothelial cells (HUVEC) model was chosen to explore the extract properties related to vascular inflammation/vasodilation. A significant reduction of both soluble Inter-Cellular Adhesion Molecule-1 (sICAM) and endothelin-1 secretion/release was induced by GSEe in HUVEC cells. A randomized, double-blind, placebo-controlled clinical study in healthy volunteers was further performed to investigate GSEe benefits. In healthy volunteers, both supplementations significantly modulated blood pressure, with a pronounced effect after GSEe tablets (300 mg/day for 16 weeks) in respect to placebo. In the male gender subgroup, no placebo effect was observed as it was for the female group. As an additional outcome, an overall GSEe positive modulation emerged on mood related to stress perception. Thus, GSEe resulted in a benefit of modulating endothelial functionality and blood pressure. It was noteworthy that GSEe relieved the perceived stress, promising new future perspectives on mood comfort.

## 1. Introduction

Cardiovascular disease (CVD) still represents a major cause of death in the world, with hypertension playing a key etiologic role in developing ischemic heart disease, cardiac and renal failure, as well as cerebrovascular diseases [1]. Dietary supplements as an alternative to pharmacological therapy for maintaining normal blood pressure are very important [2].

Proanthocyanidins are a large family of molecules existing in both monomeric and oligomeric structures up to more than 20 units. Many beneficial properties in cardiovascular, cancer, and neurological diseases as antioxidant, immunomodulatory, antidiabetic, cardio, and neuroprotective agents have long been described and recently reviewed by Rauf and colleagues [3].

Previous clinical studies in pre- and mildly hypertensive subjects [4] showed that the standardized grape seed extract (GSEe) Enovita^®^ from *Vitis vinifera* L. seeds, supplemented for a four-month period, produced beneficial cardiovascular effects. GSEe was mainly composed of oligomeric proanthocyanidins, with a low amount (5–10%) of monomeric procyanidins (catechin and epicatechin).

In order to investigate the in vitro mechanism of action of GSEe, we performed studies on eNOS (endothelial nitric oxide synthase) activation of which has been described for many plant-derived products [5] in addition to vasoconstrictor factors, such as endothelin-1, together with sVCAM (soluble Vascular Cell Adhesion Molecule) and sICAM, the cell adhesion molecules [6] linked to inflammatory processes in endothelial cells.

Due to earlier findings that some plant ingredients must be converted to the active metabolite during the digestive process [7], we investigated the effects of GSEe in vitro using GSEe as powder as well as after an in vitro simulation of digestive processes in the human stomach and small intestine.

For the in vitro experiments, human umbilical vein endothelial cells (HUVEC) were used, a cell type frequently utilized to evaluate the endothelial function.

Moreover, a clinical study evaluating the potential benefit of GSEe supplementation in mild hypertensive subjects in a double-blind, randomized, placebo-controlled parallel groups study design, was performed.

## 2. Materials and Methods

### 2.1. Reagents and Cells

Primary HUVEC were acquired by Promocell GmbH (Heidelberg, Germany); Endothelial Cell Growth Medium was supplied by Promocell GmbH).

Chemical reagents for in vitro study were purchased by Merck Life Sciences (Darmstadt, Germany).

### 2.2. Dietary Supplement

The standardized grape seed extract (GSEe) Enovita^®^ was provided by Indena (Tours, France). GSEe is a proprietary oligomeric proanthocyanidin (OPCs) rich extract made exclusively with grape seeds from white wine production. Using only water as extraction solvent, GSEe is a food-grade grape seed extract standardized to provide ≥95.0% of OPCs by spectrophotometry and a relatively low amount of flavane monomers (5.0–15.0% catechin and epicatechin, by HPLC).

For the clinical study, GSEe and the corresponding inactive matching-placebo 150 mg film-coated tablets were prepared by Indena SpA (Milan, Italy). Tablets with no active ingredient, as a blank formulation without GSEe, were used as placebo and were identical to GSEe tablets, in terms of size, shape, color, odor, and taste. GSEe and placebo film-coated tablets had similar composition in terms of inactive food-grade components (Microcrystalline cellulose, dicalcium phosphate, silicon dioxide, talc, magnesium stearate, film coating suspensions). Before releasing, the film-coated tablets were tested for appearance, average mass, uniformity of mass, HPLC content of active compounds, disintegration time, and microbiological quality.

### 2.3. In Vitro Studies

GSEe extract is rich in oligomeric proanthocyanidins. It is known that physicochemical properties of the substance class are modified in the gastrointestinal tract due to biotransformation [8]. To account for such possible impact factors, the experiments were performed with the pure extract as well as the digested solution.

A 10 mg/mL stock solution of GSEe powder was dissolved in the cell culture medium (see below) and subsequently diluted to the desired concentrations. Artificially digested GSEe solution was also prepared in order to mimic the in vivo situation, i.e., the enzymatic and pH conditions during the gastrointestinal transit, as follows: GSEe powder (150 mg) was dissolved in 120 mL aqueous mucin/pepsin solution simulating one meal; the pH was adjusted at 2.0 (by HCl) and incubated for 2 h at 37 °C under shaking. After adding pancreatin, trypsin, and bile extracts, the pH was adjusted to 7.5 and the solution was buffered by 120 mM NaCl and 5 mM KCl and incubated under shaking for 4 h at 37 °C. After incubation, the digested solution was centrifuged through a Vivaspin^®^-20 tube 10 kDa MWCO (molecular weight cut off) (VWR International; Bruchsal, Germany) to remove active enzymes and then was further diluted in culture medium as indicated in the respective assay (0.1–5% *v*/*v*) [7].

HUVEC were cultured in endothelial cell growth medium, according to the manufacturer’s recommendations. Preliminary experiments to evaluate the maximal concentration tolerated by the cells were performed by using neural red uptake assay [9].

To evaluate eNOS levels, HUVEC cells were seeded (5000/well) in a 96-well plate and treated with non-toxic concentrations of GSEe (0.1 mg/mL, 0.5 mg/mL and 1.0 mg/mL powder and 0.2%, 0,5%, and 2% digested solution) in quadruplicate; after incubation periods of 5 min and 4 h, as previously described [10,11], the cells were fixed, and phosphorylated eNOS detected by using ELISA (enzyme-linked immunosorbent assay) [Cytoglow e-NOS ELISA (Phospho-Ser 1176) ELISA kit, Assay Biotechnology Company, Inc., Fremont, CA, USA]. Vascular endothelial growth factor (VEGF) was tested at 10 ng/mL concentration as a positive control, and medium as a negative control.

For cell adhesion experiments, cells (5300/cm^2^) were treated with non-toxic GSEe concentrations (0.1 mg/mL, 0.5 mg/mL and 1.0 mg/mL powder and 0.1%, 0,5%, and 5% digested solution); after incubation periods of 4 h and 24 h, medium samples were taken and stored frozen for determination of the adhesion molecules sICAM, sVCAM, and endothelin-1. Assay was performed in two independent experiments in triplicates each. Analyses were performed for sICAM, sVCAM (IBL International, Hamburg), and endothelin-1 (RnD Systems, Minneapolis) by utilizing ELISA technique with technical duplicates for each sample according to the manufacture’s protocol.

The timelines chosen for the assays are based on the evidence [7] that for eNOS the cell reacts often very quickly to plant extracts, while the reactions to other factors such as VEGF were slower than eNOS.

sVCAM assay was included, as sVCAM is an adhesion molecule linked to the endothelial cell inflammation process, even if very low or not expressed in endothelial cells not stimulated by inflammatory enhancers [12]. Data are analyzed by one-way ANOVA and Dunnett’s multiple comparison post-test (Post-test as GSEe versus respective control for each time point and GSEe solution separately).

### 2.4. Human Study

A double-blind, randomized, placebo-controlled parallel group study was conducted in subjects with mild hypertension. The study was registered at the German Clinical Trial Register (DRKS, Germany), with the code DRKS00011745.

The study was conducted in accordance with the Principles of Good Clinical Practice and the Declaration of Helsinki and was in agreement with the regulatory and legal requirements in Germany. The protocol was approved by the Ethics Committee of Landesärztekammer Baden-Württemberg on 31 January 2017 (F-2016-115). All subjects gave their informed consent for inclusion before they participated in the study.

#### 2.4.1. Participants

80 healthy subjects (45 male and 35 female) were included, with mild elevated blood pressure (125–140 mmHg systolic pressure measured for 7 days during a run-in period) without pharmacological treatments. The age range was 40–70 years, with a Body Mass Index (BMI) range of 19–32 kg/m^2^; women were in postmenopausal period. Major exclusion criteria were polyphenol high diet (≥5 portions of vegetables or fruits per day), and intake of blood pressure-lowering medication, statins, or interfering supplements.

Subjects underwent a physical examination on their screening visit, where vital signs (blood pressure and heart rate) were measured in a sitting position after 10 min at rest to train subjects about correct blood pressure measurements. In addition, a 10-year risk Score according to SCORE Germany ≤10% (Systematic Coronary Risk Evaluation) [13] and a 12-lead ECG (electrocardiogram) was performed.

After completion of the screening procedures, subjects had to document their blood pressure over a period of one week before the first visit (V1) using standardized devices (see Section 2.4.5); if eligibility could be confirmed, subjects were randomly assigned to receive GSEe or placebo over a period of 16 weeks. Randomization was stratified for male and female subjects and was created by an external statistician by Research Randomixer software/randomizer.org. The investigational supplement and matching-placebo for the study were blinded to participants, care provider, and investigators. After 8 weeks (V2) and after 16 weeks (V3), subjects had to return to the study site for assessment of the different endpoints. In between, subjects had to document in a diary their blood pressure values, one week prior to visit 1 and in weeks 4, 8, 12, and 16 of supplementation. For a schematic overview of study design and assessment of endpoints see Figure 3.

#### 2.4.2. Dietary Intervention

Two GSEe tablets (150 mg each) were taken every day orally with sufficient water (one 5–10 min before breakfast and one 5–10 min before lunch) for 16 consequently weeks. Matching-placebo tablets were administered in the same way as GSEe. Subjects documented the date and time of intake of study products in a diary. Compliance of product intake was calculated from returned tablets and if applicable diary entries.

#### 2.4.3. Objectives

The primary objective was to determine if systolic blood pressure measured by means of a 7-day home-based measurement is modified by 16-week daily administration of GSEe, compared to placebo. The secondary objectives were evaluation of changes in diastolic blood pressure by 16-week daily administration of GSEe, compared to placebo; changes in 7-day blood pressure measurement (systolic/ diastolic) over time; changes in 24 h blood pressure as mean 24 h and mean day-time systolic/diastolic blood pressure and nocturnal dipping (systolic and diastolic) before and after 16 weeks of supplementation; endothelial functions and arterial stiffness.

Additional objectives were global assessment, stress experienced (PSQ20), quality of life (SF-12), and compliance of product intake.

#### 2.4.4. Criteria for Objectives Measurements

Systolic and diastolic pressure were measured by two methods: the first was a 7-day blood pressure measurement, in which subjects were instructed to measure their blood pressure at home with a Boso medicus PC2 device (Bosch + Sohn GmbH u. Co. KG, Jungingen Germany), once a day at the same day time, in a sitting position, after at least 10 min of rest on the left arm. Blood pressure was recorded 1 week prior to visit 1 and in weeks 4, 8, 12, and 16 of supplementation.

The second method was a 24-h blood pressure continuous measurement performed both at the beginning and at the end of the study with the same device, with a frequency of 15 min during the day and every 30 min during the night.

Endothelial function was detected by EndoPAT™ (Itamar Medical Ltd., Caesarea, Israel), an innovative, safe and non-invasive diagnostic device for vascular health assessment; EndoPAT™ is FDA-cleared (US), CE-marked (EU), and SHONIN-cleared (Japan) operator and interpreter independent instrument measuring endothelial function in the microvascular system (the 98% of the total vascular surface area [14]). The fingertip test was demonstrated to be accurate, sensitive, and reproducible [15]. The following parameters were determined, i.e., LnRHI (Logarithmized Reactive Hyperemia index) and AI75 (Augmentation Index) as follows: after assessing the basal signal, blood flow is occluded for 5 min and after deflating, the endothelial-dependent dilatation of blood vessels is measured. The RHI index was calculated by the increase of the peripheral artery tone signal (PAT) in the fingertip after 5 min of occlusion of the arm in comparison to a 5 min baseline signal. The non-occluded arm was used as a control to exclude vasodilatation or vasoconstriction induced by mental triggers. Normal values are for LnRHI > 0.51, while endothelial dysfunction is defined as LnRHI ≤ 0.51 [16]. AI75 measures arterial stiffness and is calculated via pulse waveform analysis of the PAT signal. AI75 was calculated from PAT pulses recorded at the baseline period, and the result was further normalized to a heart rate of 75 bpm. Lower AI75 values (also negative) should reflect better arterial elasticity.

The additional parameters were evaluated as follows: The Perceived Stress Questionnaire (PSQ), a validated tool to assess subjectively experienced stress independent of specific or objective occasions [17], was completed at visits 1, 2, and 3; the PSQ included four domains: worries, tension, joy, and demands. At visits 1 and 3, a quality-of-life questionnaire (SF-12, licensed from Hogrefe Verlag, Göttingen, Germany), was completed, measuring a physical and psychological score. The SF-12 questionnaire usually contains 12 items and is divided into two types of measures: the Physical Component Summary (PCS12) and the Mental Component Summary (MCS12). During study analysis, the aggregate summary scores were calculated according to von Morfeld [18] and were scored using norm-based methods. Both PCS12 and MCS12 scales were transformed to have a mean of 50 and a standard of 10 taking as a reference the US population [19].

As for the “Global Assessment”, at the end of visit 3 the subjects were asked if they felt an improvement in their general health perception due to the intervention by using three answer categories: “not at all,” “a little bit/somewhat” and “quite a bit/very much.”

#### 2.4.5. Safety

For the assessment of safety, the following were evaluated: adverse events (AE) and concomitant medication (CC), tolerability, and blood routine parameters.

During the whole study phase, subjects were asked to report all AEs or CCs in a diary. At each visit, AEs and CCs were documented in the subjects’ source documents/ CRFs. Tolerability was assessed at the end of visit 3. The subjects rated overall tolerability by selecting one of three categories: “well-tolerated,” “slightly unpleasant,” “very unpleasant.” Additional comments were recorded separately.

Blood sampling was performed prior to the study (V1) to confirm the healthy state of the participants, during the study (V2, after 8 weeks) and at the end of the study (V3, after 16 weeks). Blood routine parameters (hemogram, liver enzymes, fat status, creatinine, uric acid, and glucose) were determined in an accredited routine lab at Synlab Medizinisches Versorgungszentrum Leinfelden (Leinfelden, Germany).

#### 2.4.6. Statistical Analysis

For the human study, the primary efficacy endpoint was defined as the change of the mean value of systolic pressure from baseline (7 days before visit 1) to end of treatment (measurement during week 16 of intervention), analyzed by covariance (ANCOVA) with baseline blood pressure used as a covariate. Further secondary endpoints were evaluated exploratory applying the ANCOVA model with baseline levels as covariates or unpaired t-test where applicable for the changes between baseline and the different intervention periods. Further, a linear mixed model with repeated measures was used to evaluate changes in systolic/diastolic blood pressure based on 7-day blood pressure diaries over time (baseline, week 4, week 8, week 12, and week 16) of intervention and between groups. Additionally, the intra-group analysis (from baseline to end of treatment) was performed applying ANOVA with repeated measurements. In the case of non-normal distribution of quantitative data, non-parametric tests were applied. Non-normality was evaluated with Shapiro–Wilk test (*p* < 0.05). All statistical tests were performed two-sided, with a significance level of 0.05. The data are presented for the ITT (intention to treat) /FAS (full analysis) population. Statistical analysis and graphs were generated using GraphPad Prism (Version 5.04)/ SPSS (IBM, Version 24.0) / SAS (Version 9.3.). Continuous variables are shown as mean and 95% Confidence Interval (C.I.).

## 3. Results

### 3.1. In Vitro Cell Studies

Preliminary experiments on HUVEC cell viability were performed to select GSEe powder or digested solution concentrations to be used in further experiments (data not shown). Maximal concentrations of 1 mg/mL of powder and 5% of GSEe digest were safe and utilized for the tests.

#### 3.1.1. eNOS

**Figure 1:**
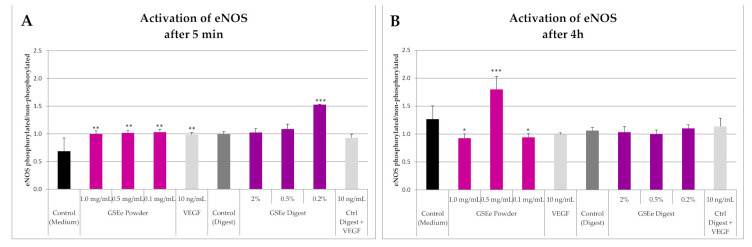
Effect of GSEe powder and digest on phosphorylated eNOS after 5 min (**A**) and 4 h (**B**). Values are given as mean ± S.D., *n* = 4. *** = *p* < 0.001; ** = *p* < 0.01; * = *p* < 0.05 calculated with one-way ANOVA and Dunnett’s post-test (GSEe versus control). GSEe: grape seed extract Enovita; eNOS: endothelial Nitric Oxide Synthase; VEGF: Vascular Endothelial Growth Factor.

An eNOS activation (about 45% increase in respect to the medium) was produced by GSEe powder at just 5 min after exposure to 0.1, 0.5, and 1 mg/mL concentration (Figure 1A); the increase was statistically significant, but not dose-related, and lower concentrations equal or below 0.01 mg/mL were ineffective (data not shown). After 4 h eNOS basal values (medium) were increased in respect to 5 min, as expected, and only 0.5 mg/mL GSEe powder produced a strong increase (about 42% increase) of phosphorylated eNOS, while a reduction was observed at 0.1 and 1 mg/mL (Figure 1B); VEGF, utilized as a control significantly increased eNOS after 5 min (Figure 1A).

Digest GSEe significantly activated (154% of control) eNOS only at the lowest concentration tested, i.e., 0.20% after 5 min incubation (Figure 1A).

#### 3.1.2. Endothelin-1, sICAM and sVCAM

**Figure 2:**
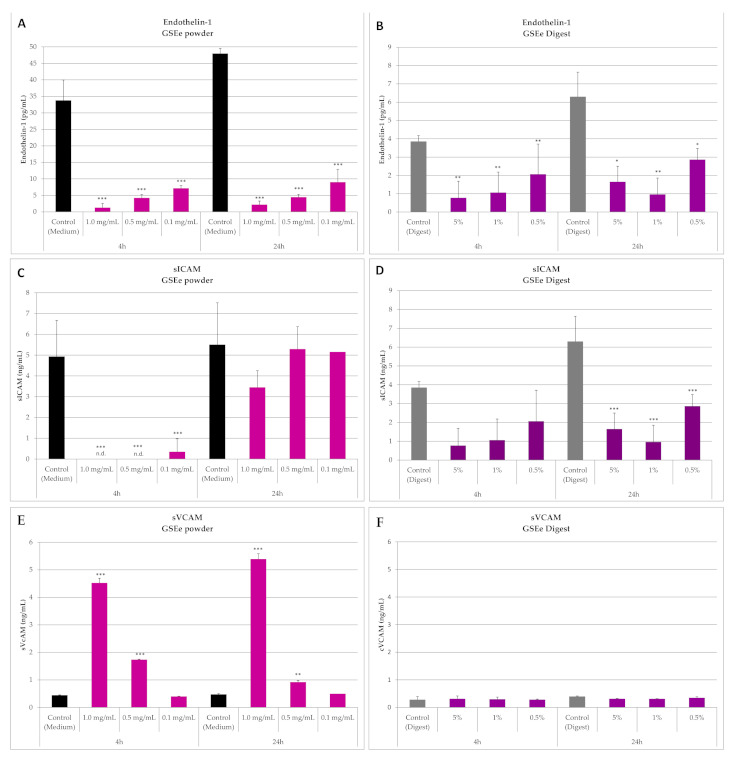
Effect of GSEe powder and GSEe digest on endothelin-1, sICAM and sVCAM secretion after 4- and 24-h incubation. In Figure 2A, Figure 2C and Figure 2E, the detection after GSEe powder administration; in Figure 2B, Figure 2D and Figure 2F the detection after GSEe digest administration. (**A**,**B**): endothelin 1; (**C**,**D**): sICAM; (**E**,**F**): sVCAM. Values are given as mean ± SD, *n* = 6, n.d. = not detectable. *** = *p* < 0.001; ** = *p* < 0.01; * = *p* < 0.05 calculated with one-way ANOVA and Dunnett’s post-test for each time point (GSEe versus control). GSEe: grape seed extract Enovita; sICAM: soluble Inter-Cellular Adhesion Molecule-1; sVCAM: soluble Vascular Cell Adhesion Molecule.

When GSEe powder was added to the HUVEC cells, a strong significant dose-dependent reduction in endothelin-1 secretion (from 33.8 ± 6.2 pg/mL to 1.3 ± 1.2 pg/mL) was observed at just 4 h; a similar profile was shown at 24 h (Figure 2A). A decrease in endothelin-1 levels was still evident at all the concentrations tested, with a 50% reduction at 0.01 mg/mL (data not shown).

A significant reduction (80% and 74%, respectively) of endothelin-1 secretion after 4h and 24 h incubation was also observed after a 5% GSEe digest treatment. A significant reduction was also evident for 0.5% and 1% concentrations (Figure 2B).

sICAM secretion was low in untreated cells; when GSEe powder was added, a complete (100%) reduction of sICAM secretion was induced by 0.5 and 1 mg/mL concentrations after 4 h of exposure (Figure 2C). When GSEe digest was added, a dose-dependent reduction (47–80% and 55–74%) of sICAM secretion was observed at both 4 and 24 h time points (Figure 2D).

Incubation with GSEe powder increased sVCAM secretion in a dose-dependent manner at 4 and 24 h, as shown in Figure 2E). It is most likely that this upregulation effect is triggered by the remaining small powder particles since the other inflammatory markers were not elevated. In addition, the digest did not affect sVCAM secretion at any tested concentration (Figure 2F).

### 3.2. Human Study

**Figure 3:**
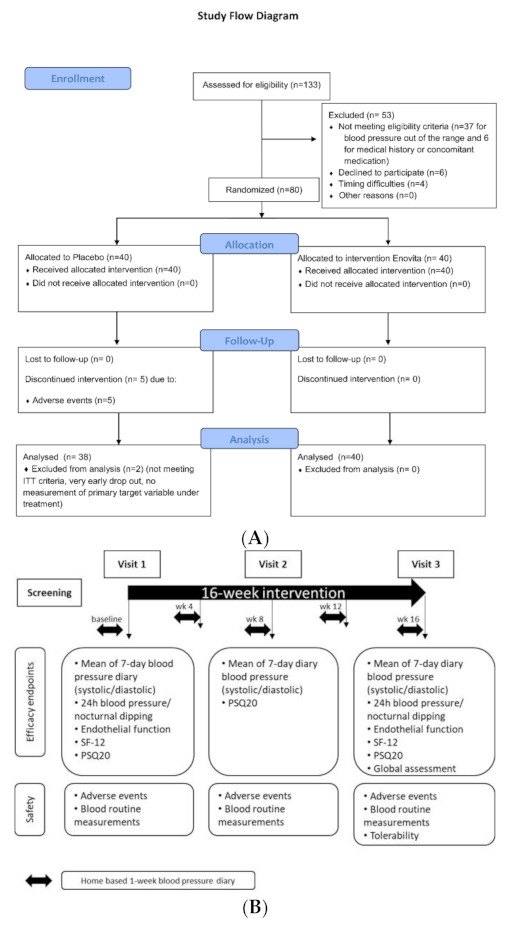
(**A**) Study flow diagram. (**B**) Schematic overview of study design and assessment of endpoints; SF-12: 12-Item Short Form Health Survey; PSQ20: Perceived stress questionnaire.

The study flow diagram has been reported as Figure 3. After the assessment for eligibility of 133 healthy subjects, 80 subjects of both genders were enrolled and randomized to receive GSEe or placebo supplementation. 2 subjects in the placebo group were excluded from the analysis because they did not meet ITT (intention to treat) criteria, as reported in the following study flow chart (Figure 3A). Furthermore, in Figure 3B a schematic overview of study design and assessment of endpoints is represented.

Male subjects were 54.3 (95% C.I. 47.5–60.5) years old while women were significantly older with 59.9 (95% C.I. 56.0–63.5) due to the inclusion criteria of the menopausal condition. No differences in BMI were observed between males and females. The distribution by gender in the placebo and supplemented groups was homogeneous. Baseline conditions of the study groups are summarized in following Table 1.

A significant reduction of systolic and diastolic blood pressure was seen in both study groups (placebo *p* = 0.0086 (systolic) (Figure S1); *p* = 0.0182 (diastolic); GSEe: *p* < 0.0001 (systolic) (Figure S1); *p* = 0.0005 (diastolic) whereas the blood pressure decrease was slightly more pronounced during intervention with GSEe for systolic and diastolic. Evaluating the blood pressure between and within groups using a linear mixed model with repeated measures considering age, gender, and BMI as covariates confirmed that there was no product effect (systolic BP: *p* = 0.8236, diastolic BP: *p* = 0.3189) but a significant time effect *p* < 0.0001.

**Figure 4:**
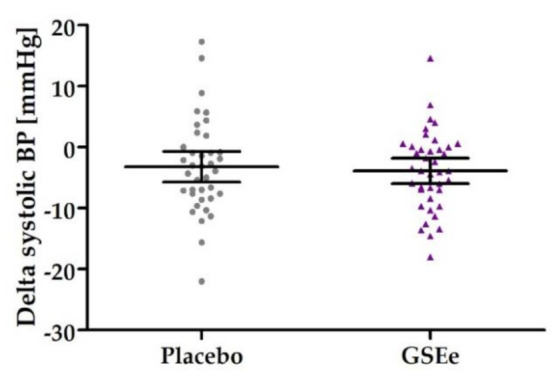
Primary endpoint as distribution of systolic blood pressure in placebo and GSEe. Distribution is expressed as changes of systolic blood pressure in mmHg between the end of supplementation with placebo (in grey) or GSEe (in purple) and baseline measurements. Scatter diagram with mean ± 95 % C.I.

The primary efficacy endpoint, defined as the change of the mean value of systolic blood pressure from baseline (7 days before visit 1) to end of treatment (16 weeks), did not show statistic differences between placebo and GSEe group for the systolic (*p* = 0.7197) and diastolic mean values (diastolic BP *p* = 0.2001). As shown in Figure 4, systolic blood pressure was reduced by a magnitude of 3.21 (95% C.I. 5.74–0.68) mmHg in the placebo group and 3.90 (95% C.I. 5.97–1.83) mmHg in the GSEe group.

**Figure 5:**
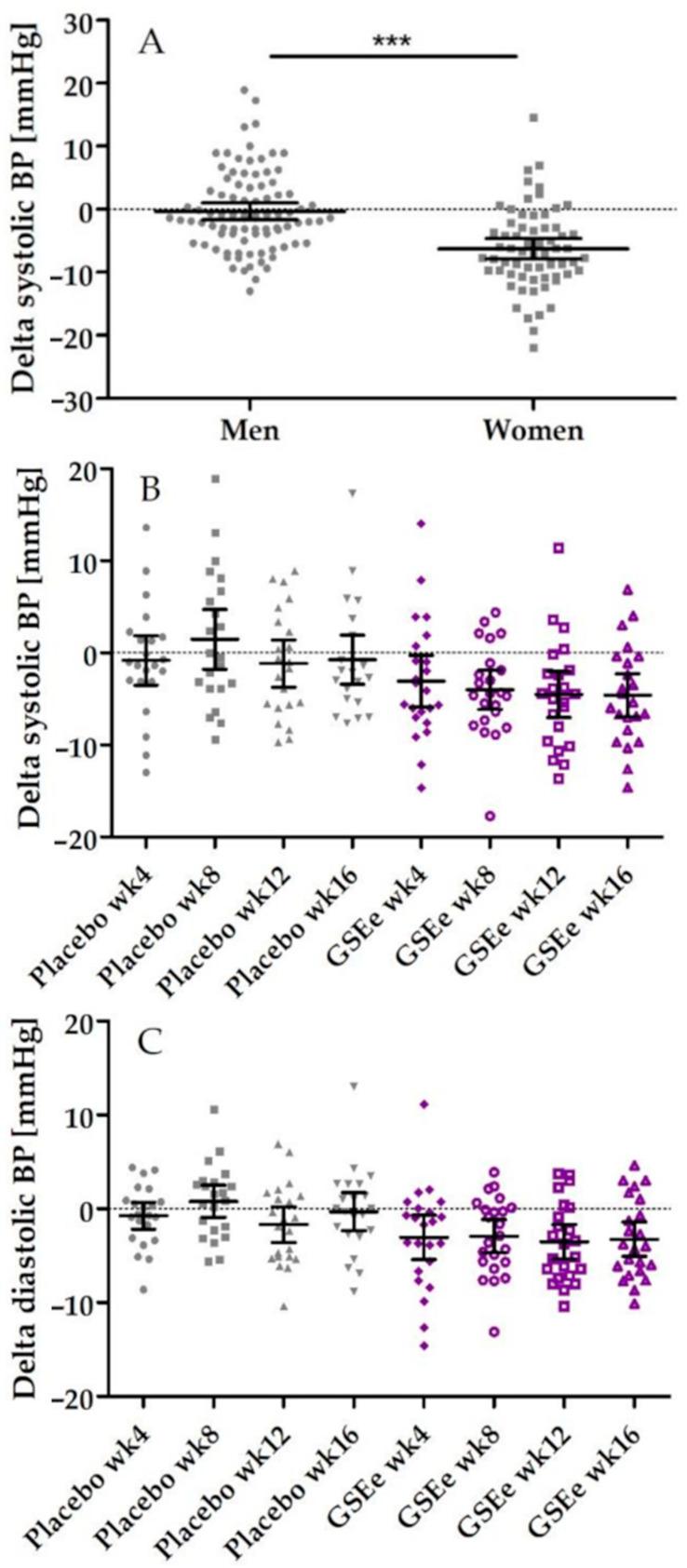
Distribution of delta changes of blood pressure interactions between gender and supplementation. (**A**). Distribution of delta change of 7-day diary of systolic blood pressure measurement after 4, 8, 12, and 16 weeks in comparison to baseline assessment in men and women during placebo intake summarized for all assessment time points. (**B**). Distribution of delta changes of 7-day diary of systolic blood pressure assessment in men in comparison to baseline assessment. (**C**). Distribution of delta changes 7-day diastolic blood pressure in men in comparison to baseline assessment. Grey: placebo, purple: GSEe group [in mmHg]. All data pooled for 4-week, 8-week, 12-week and 16-week assessment (panels (**B**,**C**)). Scatter diagram with mean ± 95 % C.I.; *** = *p* < 0.0001.

Further investigations indicated for the primary endpoint a significant interaction between gender and product. Therefore, data were additionally evaluated by gender. As shown in Figure 5A men showed only a low response to placebo treatment, whereas the placebo response in women was quite distinct and significantly higher than in men (average placebo effect men: −0.31 mmHg (95% C.I.: −1.65 to 1.03); average placebo effect women: −6.27 (95% C.I.: −7.90 to −4.64) *p* < 0.0001).

Linear mixed models indicated for men group a GSEe effect by trend (*p* = 0.0701) controlling for the time (*p* = 0.0072) and an interaction of GSEe × time (*p* = 0.0510). To investigate the single time points, pair-wise tests of the delta levels by gender were performed and results are summarized in Table 2.

Significant differences between placebo and GSEe treatment were observed in men after 8 weeks and 16 weeks, leading to a statistically significant systolic and diastolic blood pressure reduction (Figure 5B,C). In particular, a reduction of systolic blood pressure of −4.6 mmHg (95% C.I.: −6.9 to −2.3) and diastolic blood pressure of −3.2 mmHg (95% C.I.: −5.1 to −1.4) was observed in men after 16 weeks of intervention.

In women, no superiority of GSEe on blood pressure reduction in comparison with placebo was seen (data not shown). Indeed, the mean blood pressure reduction in women was even more pronounced under placebo in comparison to GSEe but only after 4 weeks of supplementation (*p* = 0.0057) (Table 2).

Next to the home-based blood pressure measurements under resting conditions, a 24 h blood pressure measurement was performed at the beginning and end of the intervention. Interestingly, in both subgroups of women and men the slight reduction of systolic blood pressure with a benefit towards GSEe was confirmed, which was even more pronounced in women (Delta change of mean day pressure: GSEe men: −1.6 mmHg vs. GSEe women: −2.34 mmHg). The benefit was also descriptively seen in women for the endpoint mean 24 BP with a reduction of −3.81 mmHg (95% C.I.: −7.79 to 0.16) after intake of GSEe in comparison to placebo intake (increase 1.09 (95% C.I.: −3.25 to 5.43) of systolic blood pressure. Furthermore, nocturnal dipping increased significantly in comparison to placebo in women (+2.77 vs. −2.52, respectively; *p* = 0.0328). The physiological decrease in nocturnal blood pressure relative to daytime blood pressure is referred to as nocturnal dipping. Prospective studies have shown that each 5% attenuation in the nocturnal BP decline conferred a ∼20% increase in the risk of cardiovascular mortality in the general or hypertensive population [20,21]. Thus, an increase of nocturnal dipping during GSEe supplementation is a positive outcome associated with a reduced risk of cardiovascular disease.

The endothelial function measurement identified that 13.2% of the placebo group and 32.5% of GSEe had lnRHI levels ≤ 0.51 indicating endothelial dysfunction. Furthermore, as expected, a significant correlation was observed between age and AI75 (r = 0.4395, *p* < 0.0001) (Figure S2). However, neither for LnRHI nor AI75 were observed significant changes between and within groups after 4 months of intervention. The values obtained showed high intra-individual variability in both study groups (data not shown). In both study groups, no changes were observed over the 4-month intervention period.

Additional objectives were analyzed in order to evaluate global assessment, perceived stress, and physical and mental components, as planned in the approved study Protocol. Perceived stress and mental components can especially impact blood pressure. Furthermore, it is known that polyphenols can also modulate mood disorders via the gut-brain axis [22].

**Figure 6:**
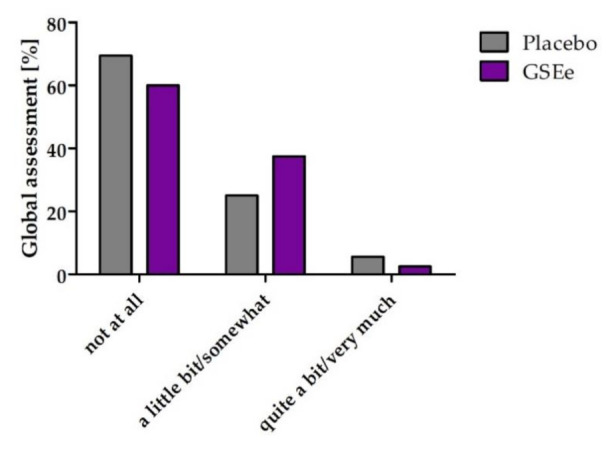
Global assessment 16 weeks after (V3), as general health perception due to intervention. Global assessment in percentage of GSEe (in purple) vs. placebo (in grey) evaluated by three answer categories: “not at all”, “a little bit/somewhat” and “quite a bit/very much”.

The ratio of subjects experiencing an overall improvement of their general health perception was slightly higher during GSEe supplementation in comparison to the placebo treatment without reaching statistical significance (*p* = 0.4387) (Figure 6). In general, the frequency of women reporting an overall improvement of health perception was higher than in men. However, in both genders, the frequency of subjects with improvements was higher in the group of GSEe (men: 26.1% GSEe vs. 19.0% placebo; women: 58.8% GSEe vs. 46.7% placebo).

At baseline (Visit 1), no statistical differences among groups were observed, by investigating the 4 domains of the PSC perceived stress questionnaire, i.e., worries, tension, joy, and demands with 5 items each. A significant reduction of worries in the GSEe group (*p* = 0.0199, Table 3) was observed. Comparing the delta changes of V3-V1 of worries between GSEe group and placebo group, a significant difference for the worries subscore (*p* = 0.0252) was observed (Table 3). For the other scores, there was also a slight reduction in tension and demands and increase of joy (data not shown).

The observations in the GSEe group were confirmed in men and women. In both subgroups, no changes or rather a very minor worsening, were observed between the beginning and end of the intervention in the placebo groups, but a distinct reduction of the subscale PSQ worries under GSEe supplementation which was more pronounced in women than in men (women: V1: 21.2 ± 16.0, V3: 17.3 ± 18.4, *p* = 0.0327; men: V1: 13.0 ± 13.4; V3: 11.0 ± 13.7, *p* = 0.3679); Differences between placebo and GSEe (women: *p* = 0.0629; men: *p* = 0.2202). Worth mentioning, women had on average significantly higher PSQ worries levels (women total group V1: 23.6 ± 14.9; men total group V1: 12.4 ± 12.6; *p* < 0.0008) underlining that such stress perceptions affect women to a greater extent.

Interestingly, regarding the score analysis for SF 2 questionnaires, MCS12 scores increased in the GSEe group by trend between visit 1 and visit 3 (*p* = 0.0593), while in the placebo group the MCS12 slightly decreased (Table 4). Comparing the delta changes between placebo and GSEe differences by trend between groups for MCS12 were observed (*p* = 0.0733). For the PCS12 scores, no differences were observed between and within groups.

The responses of the mental summary scores are supported in both genders with an improvement under the intake of GSEe, whereas the extent was more pronounced in women (change by a trend in women, *p* = 0.0832). No improvement or slight worsening was observed under the placebo intervention in men and women.

Importantly, GSEe administration was very well tolerated. In the placebo group, two Serious Adverse Events (SAEs) were reported and occurred accompanied with hospitalization, but no SAEs were reported in GSEe group. None of the AEs observed were related to the treatment.

No safety issues were identified checking the blood routine parameters. Compliance of supplementation intake was very good in both study groups.

## 4. Discussion

In the present study, we report the ability of a food-grade highly standardized grape seed extract GSEe to modulate blood pressure status in humans, and surprisingly, its benefit on mood related to stress perception. Deep preclinical research was also conducted to explore the potential mechanism of action of GSEe in blood pressure optimization.

Hypertension condition, responsible for 7.5 million deaths/year [17], is a serious medical status that needs great consideration. It is reported that in Europe more than 150 million people are affected by hypertension, with a predicted increase by 15% to 20% by 2025 [18]. To prevent complications due to a persistent hypertensive status, which can lead to serious cardiovascular problems causing heart attack and ischemia, or CVD such as stroke or paralysis, antihypertensive treatments are used. Together with a pharmacological approach, and especially in prevention and in the first phase of disease, natural products can be an important way to block the disease progression [1].

Beneficial properties of grape seed extracts shown by several reports are many, including antioxidant [23,24], anti-inflammatory, neuro-protectant, and free radical scavenging abilities [25], as anti-proliferative agent [26] or to counteract cellular toxicity by chemotherapeutic agents [27], as well as in Alzheimer’s animal models [28] as neuroprotective, and in CVD where vasodilating [29] and cardioprotective effects are displayed [30].

In the present paper, we showed evidence that GSEe in vitro on HUVEC cells, widely utilized human-derived primary endothelial cells, produced a significant reduction of both sICAM and endothelin-1 secretion/release. Those effects, together with a significant activation of the vasodilating agent eNOS [31], support the beneficial effects of GSEe on vasodilatation and inflammation, as shown in other previous studies by proanthocyanidins [29,32,33,34]. The effect of GSEe on sVCAM appeared less clear, but since sVCAM is normally very low or not expressed in endothelial cells [33], it should be investigated better with other experiments such as inflammatory-induced models. It is hypothesized, that the findings of elevated sVCAM expression were caused by particle stress [7]. For the digest, the solution was filtered prior to use in the model.

Data obtained on HUVEC are of particular interest, because endothelial dysfunction is regarded as an additional independent risk factor for CVD, and it is developed in the early stages of atherosclerosis [12]. The promising data obtained in vitro led us to investigate if the GSEe supplementation would be beneficial to mild hypertensive subjects.

A 4-month administration with GSEe (300 mg/day) in pre- and mildly hypertensive subjects had already been demonstrated to be effective in normalizing blood pressure in 93% of the supplemented group [4]. Furthermore, meta-analysis of ten randomized controlled clinical trials with daily grape polyphenol supplementation at different dosages [35] confirmed a significant reduction of systolic blood pressure compared to control group, with no significant reduction in diastolic pressure. Another meta-analysis data on 16 randomized controlled trials performed by Zhang et al. [36] suggested a positive effect on blood pressure by GSE.

Results from the present randomized, double-blind, placebo-controlled study in mild-hypertensive volunteers indicated that the systolic blood pressure lowering effects were comparable between study groups, with no amelioration seen by 16 weeks’ GSEe supplementation.

However, further evaluations for the primary endpoint indicated a significant interaction between product x gender, so when the subgroup analysis by gender was performed, a product effect by trend (*p* = 0.0701) was observed in men with significant differences between placebo and GSEe supplementation after 8 weeks and 16 weeks for systolic and diastolic pressure indicated in the pair-wise tests.

On the contrary in women, who showed a high response to placebo treatment, the beneficial activity of GSEe possibly was hidden by placebo. This interpretation is supported by the significant effects seen in nocturnal dipping in women, as these measures during the night might be less influenced by placebo effects. However, one must keep in mind that the night measurements could impact the measurement results due to sleep interruption posing a high burden to subjects. There might be individual differences in the tolerance of the measurement in men and women.

Gender differences are already described in the literature in terms of response to placebo in clinical studies [36], and those differences could take into account the lack of achievement of the primary endpoint of the study of women.

The major proportion of women vs. men in the present study in respect to the previously published clinical study of Belcaro [4] using GSEe at the same dose and time (300 mg die for 3 months) would possibly explain the differences in the final results. Furthermore, in the present study, the age range was 40–70 years with men being significantly younger in comparison to women because of the inclusion criterion of post-menopause for women, while in Belcaro’s study the range of age is narrower, i.e., 45–55 years. Belcaro’s study of included subjects with stage I hypertension (140–159 mm Hg systolic pressure), while in the present study only pre-hypertensive (120–140 mm Hg) subjects were recruited. This hypothesis is also supported by the meta-analysis of Zhang et al. 2016 [36], who concluded that the impact of grape seed extract on blood pressure was more obvious in younger (<50 years) or obese subjects, as well as in patients with metabolic disorders.

The biological mechanisms underlying the benefit of blood pressure reduction so far remain largely speculative [36]. It is widely recognized that grape seed extract contains antioxidants that can help to prevent cell damage caused by free radicals. Furthermore, experimental data have indicated that grape seed extract could lead to an endothelium-dependent relaxation in rabbit aorta [37]. Additionally, enhancement of expression and activity of endothelial nitric oxide synthase in human umbilical vein endothelial cells are discussed [38]. This mechanism is supported by the present in vitro findings on eNOS and reduction of Endothelin-1. In the study of Belcaro et al. [4] the microcirculation state was evaluated with a laser Doppler flowmetry which confirmed a significant increase. However, the endothelial function in the current study was measured with another method, i.e., the noninvasive EndoPAT™ 2000. The parameter lnRHI collected is reflecting the responsiveness of the endothelial cells on 5 min ischemia/ hypoxia by finger plethysmography. With EndoPAT™ the endothelial function of the microcirculatory system is assessed, and over the study period, no changes between and within groups were identified. Even if the two techniques (laser Doppler flowmetry and EndoPAT™) are widely utilized and very well characterized, a correlation of the results obtained has not always been verified [39]. This is a limit of the present study, even if data obtained are in line with those of Park et al. [40], who investigated a grape seed extract of 300 mg over a 6-week parallel study using ultrasound technology in the brachial artery to measure endothelial function. Further, the researcher did not measure an impact on flow-mediated dilatation, despite the grape seed extract significantly reducing systolic blood pressure (SBP) by 5.6% and diastolic blood pressure (DBP) by 4.7% after 6 weeks of intervention period [40].

Despite the significant mechanistic findings documented with in vitro research experiments, it seems that the endpoint endothelial function is complex and influenced by diverse triggers (e.g., adrenalin and other physiological factors) that the effectiveness of GSEe might be less obvious in the presence of these factors. Data indicated high intra-individual variability despite high standardization of the measurements.

The low response rate to the global assessment is not surprising, as slightly elevated blood pressure normally doesn’t cause any noticeable symptoms, and thus an improvement is not accompanied by noticeable changes. Nevertheless, the findings are in line with the interesting results on stress perception and a mental component score, considering that stress is a well-known trigger or intensifier of elevated blood pressure [41,42,43].

Of note is a statistically significant reduction of worries, as well as an improvement of the mental component score by trend, which could be noticed after GSEe supplementation, indicating that a beneficial effect on stress and mood is associated with the GSEe supplementation. Those data are in agreement with a reduction of physical and psychological symptoms such as anxiety, evaluated according to the HADS questionnaire [44] observed in menopausal women supplemented by a grape seed proanthocyanidin extract [45]: in that clinical study, a reduction of systolic pressure was also demonstrated.

The positive changes might be attributed to the polyphenol content in GSEe. Polyphenols have been strongly associated with higher cognitive function, better mood, and protective effects against various brain diseases in literature [46,47,48]. In a study with healthy subjects, purple grape juice significantly improved reaction time on a composite attention measure and increased calm ratings when compared to placebo [48].

Future perspectives can be planned in order to explore in-depth the potential of GSEe in the stress relaxing field, considering the positive unexpected results observed. Several efforts are ongoing on a bio-based productive process for a GSEe with an equivalent phytochemical profile, in order to support the environmental sustainability related to grape seeds ingredients.

## 5. Conclusions

Finally, we can conclude that GSEe, the highly standardized extract in polyphenols of *Vitis vinifera* L., is effective in modulating endothelial functionality and blood pressure, especially in men. A potential benefit in mood related to stress perception was also observed.

## Figures and Tables

**Table 1:** Population characteristics, vital signs, and blood markers at baseline.

	Placebo	GSEe
N	38	40
Age [years]	56.9 (54.6–59.3)	56.4 (53.9–58.9)
BMI [kg/m^2^]	26.1 (25.1–27.1)	25.2 (24.1–26.3)
Known familiar incidence of elevated blood pressure	45%	43%
Frequency [%] men / women	57.9% / 42.1%	57.5% / 42.5%
10-year risk Score according to SCORE Germany ≤5%; Frequency [%]	87%	85%
Systolic blood pressure [mmHg]	134.1 (132.8–135.4)	134.6 (133.2–136.0)
Diastolic blood pressure [mmHg]	82.3 (80.3–84.4)	83.7 (81.8–85.7)
Cholesterol [mg/dL]	229 (217–241)	231 (220–241)
LDL-cholesterol [mg/dL]	142 (132–152)	141 (132–150)
GPT [U/L]	29 (25–33)	26 (23–28)
GOT [U/L]	22 (20–24)	20 (19–22)
Glucose [mg/dL]	94 (91–97)	91 (88–94)

Values are expressed as mean; in parenthesis 95% confidence interval as for the text. BMI: Body Mass Index; LDL: low-density lipoprotein, GPT: glutamate pyruvic transaminase, GOT: glutamic oxaloacetic transaminase.

**Table 2:** Summary of significance levels of pair wise comparisons by gender for baseline-corrected systolic and diastolic blood pressure changes from 7-day diary.

	Men ^1^				Women ^2^			
	Estimated Mean Difference	95% C.I. of Mean Difference Lower upper		*p* Value	Estimated Mean Difference	95% C.I. of mean Difference Lower Upper		*p* Value
**Systolic Pressure**								
After 4 weeks	−2.19	−5.97	1.59	0.2489	4.76	1.49	8.03	0.0057 *
After 8 weeks	−5.36	−9.01	−1.70	0.0051 *	0.98	−3.51	5.47	0.6599
After 12 weeks	−3.27	−6.71	0.17	0.0619 °	0.98	−3.36	5.33	0.6469
After 16 weeks	−3.76	−7.16	−0.37	0.0306 *	3.73	−2.22	9.67	0.2103
**Diastolic Pressure**								
After 4 weeks	−2.15	−4.90	0.61	0.1234	2.60	−0.23	5.42	0.0702 °
After 8 weeks	−3.59	−6.04	−1.14	0.0051 *	0.56	−2.82	3.95	0.7363
After 12 weeks	−1.77	−4.38	0.85	0.1795	0.22	−3.33	3.77	0.8998
After 16 weeks	−2.98	−5.70	−0.27	0.0320 *	0.69	−2.97	4.35	0.7046

Change of 7-day blood pressure measurement over time compared to placebo. *p*-values are reported from ANCOVA with baseline blood pressure as covariate. 95% confidence interval (C.I.). ^1^ Men, *n* = 22 placebo, *n* = 23 GSEe; ^2^ women, *n* = 16 placebo, *n* = 17 GSEe. * statistical significance. ° near significance. The blood pressure was measured during seven days at home before starting supplementation, and in week 4, 8, 12, and 16 of GSEe supplementation.

**Table 3:** Perceived Stress Questionnaire (PSQ20) worries score.

PSQ20 Worries Score	Placebo V1	Placebo V2	Placebo V3	GSEe	GSEe V2	GSEe V3
Mean	17.9	14.9	18.4	16.5	14.7	13.7 *
Std Deviation	14.5	15.5	16.3	14.9	14.6	16.0
Lower 95% C.I.	13.1	9.8	13.1	11.7	10.0	8.6
Upper 95% C.I.	22.7	20.0	23.8	21.3	19.3	18.8

Number of values are 38 for placebo group at baseline (V1), after 8 weeks (V2) and after 16 weeks (V3), and 40 for GSEe groups at V1, V2, and V3; * *p* < 0.05, statistically different applying Friedman test. Std: standard; C.I.: confidence interval.

**Table 4:** Descriptive statistics of delta MCS12 and PCS12 score between end of intervention and baseline.

SF-12	Placebo PCS12	GSEe PCS12	Placebo MCS12	GSEe MCS12
Mean	−0.15	0.07	−1.71	1.32 *
Std Deviation	6.99	3.16	7.77	4.67
Lower 95% C.I.	−2.44	−0.94	−4.27	0.18
Upper 95% C.I.	2.15	1.08	0.84	2.81

Descriptive statistics of delta MCS and PCS score. Physical Component Summary (PCS12) and the Mental Component Summary (MCS12) were evaluated as difference between end of intervention and baseline; * difference by trend GSEe vs. placebo (*p* = 0.0733). Std: standard; C.I.: confidence interval.

## Data Availability

The data presented in this study are available on request from the corresponding author. The data are not publicly available due to privacy and ethical reasons.

## Supplementary Materials

The following are available online at https://www.mdpi.com/2072-6643/13/2/654/s1, Figure S1: Distribution of systolic blood pressure from 7-day diary in placebo (A, in grey) and in GSEe (B, in purple ♦) group [mmHg]. Scatter diagram with mean ± 95 % CI. Figure S2: Correlation of Age with augmentation index AI75 of all subjects at baseline measurement. Pearson *r* = 0.4395; *p* < 0.0001.
