# Supplementary material for: Grape Seed Extract Positively Modulates Blood Pressure and Perceived Stress: A Randomized, Double-Blind, Placebo-Controlled Study in Healthy Volunteers
Source: Nutrients. 2021 Feb 17;13(2):654. doi: 10.3390/nu13020654 (PMC7922661; doi:10.3390/nu13020654)
Supplement: Supplementary file 1 [file nutrients-13-00654-s001.pdf]

nutrients

Article

Grape seed extract positively modulates blood pressure and perceived stress: a randomized, double-blind, placebo controlled study in healthy volunteers

Christiane Schön[1,*], Pietro Allegrini[2], Karin Engelhart-Jentzsch[1], Antonella Riva[2], Giovanna Petrangolini[2]

[1] BioTeSys GmbH, Schelztorstr. 54-56, 73728 Esslingen, Germany; c.schoen@biotesys.de (C.S.), k.engelhart@biotesys.de (K.E.)

[2] Research and Development Department, Indena SpA, 20139 Milan, Italy; pietro.allegrini@indena.com (P.A.), antonella.riva@indena.com (A.R.), giovanna.petrangolini@indena.com (G.P.)

* Correspondence: c.schoen@biotesys; Phone: +49 (0) 711/31 05 71-45

Received: date; Accepted: date; Published: date

Supplementary Figures

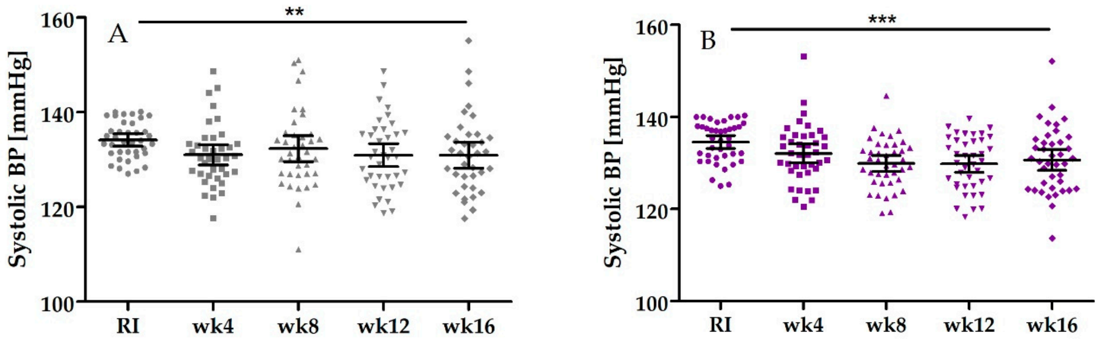

Figure S1. Distribution of systolic blood pressure from 7-day diary in placebo (A, in grey) and in GSEe (B, in purple ♦) group [mmHg]. Scatter diagram with mean ±95 % CI.

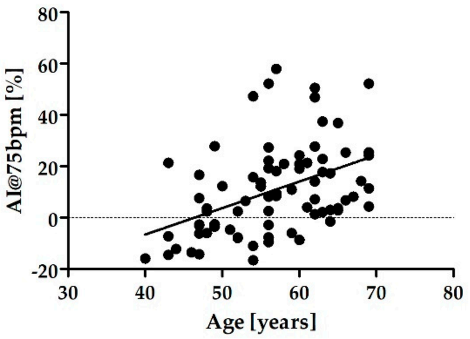

Figure S2. Correlation of Age with augmentation index AI75 of all subjects at baseline measurement. Pearson r=0.4395; p<0.0001
